# Development and validation of an unsafe behavior checklist for workers

**DOI:** 10.1371/journal.pone.0314571

**Published:** 2024-11-27

**Authors:** Myung-Hee Kim, Jin-Hyuk Hong, Byung-Jo Moon, Woo-Je Lee, Jin-Woo Jung

**Affiliations:** Department of Safety Engineering, Seoul National University of Science and Technology, Seoul, Republic of Korea; PLOS, UNITED KINGDOM OF GREAT BRITAIN AND NORTHERN IRELAND

## Abstract

The purpose of this study is to develop and validate a simple unsafe behavior checklist to assess the extent of unsafe behavior among workers in an industrial setting. The checklist was developed using Focus Group Interview (FGI) and Analytical Hierarchy Process (AHP) analysis methodology. Preliminary testing was conducted with an unspecified sample and the correlation between unsafe behavior and accident experience was validated. The analyzed and compared the correlation between the type of unsafe behavior and accident experience and demonstrated the suitability of the unsafe behavior checklist. A risk sensitivity and risk-taking checklist was employed to identify five types of unsafe behavior. This study concludes that the unsafe behavior checklist can be used to predict accident concerns among workers with high types of dangerous behavior. This can help improve unsafe behavior and reduce the frequency of incidents. Therefore, the “Unsafe behavior Checklist” developed in this study can be used as an early indicator of the extent to which workers are aware of dangers in industrial workplaces in order to prevent accidents.

## 1. Introduction

An examination of the underlying causes of workers’ exposure to industrial accidents in reveals two broad categories of accidents: those involving material factors, which are caused by mechanical defects, and those involving human factors, which are caused by workers’ carelessness or mistakes. Material factors encompass unsafe conditions, such as inadequate maintenance and a poor environment, while human factors involve the unsafe behavior of workers.

A person’s unsafe behavior is a factor that can directly cause an accident; therefore, it becomes a human factor., unsafe behavior includes any actions that create dangerous conditions, such as working at an unsafe speed, not operating protective devices, not attaching caution signs, not working with prepared tools or equipment, and not wearing protective equipment.

Most of the accidents that occur in industrial workplaces are caused by workers’ unsafe behavior [1, 2]. Hence, the reduction of industrial disasters and accidents is largely dependent on the improvement of workers’ safety behavior [3]. Unsafe behavior can negatively impact the risk, safety culture, and overall production of workers [4, 5].

Therefore, numerous researchers have corroborated the concept that accidents are caused by dangerous work behavior [6, 7].

Studies have examined the factors that trigger unsafe behavior and their impact on unsafe acts and accidents, with the aim of improving safety behavior [1, 8, 9].

Moreover, researchers have analyzed the relationship between unsafe behavior and individual causes and the cognitive failures that lead to the choice of unsafe behavior as well as the factors that increase safe behavior or decrease unsafe behavior [10, 11].

Behavior-Based Safety (BBS) [12, 13] refers to interventions that aim to modify specific behaviors by applying behavioral psychology principles [14].

Examination of the existing research on unsafe behavior, by discipline, reveals positive behavioral supports and checklists for preventing problematic behaviors among students with behavioral problems [15, 16].

Since risk behaviors in youth cannot be prevented, studies have developed measures to examine risk behaviors [17]. Checklist scales have been developed to identify risks to children [18], as have predictive tools that comprehensively consider and evaluate potential outcomes to prevent criminals from causing harm [19].

Furthermore, studies have been conducted on behaviors and dangerous driving, and dangerous driving styles have been differentiated in research [20, 21].

Additionally, a study developed a risky behaviors checklist as a means of identifying unsafe performance among nurses to enable the management of potentially problematic nurses [22].

Considering this, individualized assessments are needed for effective interventions for a variety of risky behaviors [23].

However, in Korea, while a safe driving behavior checklist exists for elderly drivers over 60 years old [24], most checklists focus on identifying workplace hazards [25, 26]. Furthermore, safety behavior checklists in the construction industry are primarily centered on manager-led observation and evaluation of workers [27, 28].

To date, most studies have focused on analyzing unsafe behavior through safety perceptions and the causes of workers’ unsafe behavior.

However, instead of investigating the workforce as a whole, this study concentrates on examining the types of unsafe behavior among individual workers.

In Korea, there is a lack of unsafe behavior checklists that allow workers to self-assess their own actions. Therefore, this study aims to develop and validate a checklist that enables individuals to identify their unsafe behaviors.

This type of assessment is crucial for the differential diagnostic assessment of an individual’s risk tolerance in an emergency [29], since it is important to distinguish between types of unsafe behavior based on the individual engaging in the behavior.

Nevertheless, checklists are lacking for dangerous worker behaviors in the workplace, as are indicators to differentiate danger types. Therefore, in this study, we propose a unsafe behavior checklist that can be used to identify unsafe behavior among workers.

## 2. Method

A series of rigorous steps was employed to develop a checklist to help determine the type of unsafe behavior.

### 2.1 Development of unsafe behavior checklist

#### 2.1.1 Unsafe behavior checklist scale covering risk sensitivity and risk-taking

The causes of unsafe behavior can be divided into mistakes and violations. Mistakes are unsafe behaviors in which awareness of the risk is lacking, while violations are intentional acts that are carried out with the acknowledgment of the risk.

Therefore, the scale of the unsafe behavior checklist is based on two key factors: risk sensitivity, which is the inability to perceive risk, and risk-taking, which is the intentional violation of a rule despite knowledge of the associated danger [30, 31].

Risk sensitivity refers to the ability to intuitively identify what is risky and how certain actions could lead to dangerous situations as well as the ability to sensitively recognize the degree of risk [4]. The ability to perceive that something is a risk is called “risk-sensitivity” [30, 32].

Conversely, risk-taking indicates “the type of danger that is considered acceptable” [33, 34]. The individual’s risk tolerance is one of the main reasons for unsafe behavior in the workplace [33].

Therefore, the unsafe behavior scale consists of risk sensitivity and risk-taking.

## 2.1.2 Organization of checklist questions

The questionnaire for this study was developed based on the checklist proposed by Reason (2017) [35].

Responses are indicated as "yes" or "no". To minimize response bias and enhance data reliability, both positive and negative response options were included for each item, allowing respondents to accurately reflect their own status [36].

Previous studies on unsafe behavior and risk sensitivity were reviewed to construct the risk-sensitivity and risk-taking items [1, 6, 22, 37–41]. These items were adapted for this study’s goal, and the factors that lead to unsafe behavior and its causes were included.

In addition, the safety check sheet of the Korea Occupational Safety and Health Agency was reviewed [42], along with the relevant literature on the prevention of occupational accidents. The survey questions were then modified and supplemented to adapt them for the purposes of this study [6, 32, 37–41, 43, 44].

To ensure the reliability of the constructed items, focus group interviews (FGI) were conducted with five safety experts with more than 10 years of experience, and 48 checklist items were derived.

The research objectives were explained, and the drafted questionnaire was initially presented to a group of experts to gather their feedback. Subsequently, focus group interviews were conducted, and the questionnaire was meticulously reviewed, revised, and refined based on the expert opinions and the focus group discussions. Items deemed unsuitable were removed from the unsafe behavior checklist.

Recent analysis of industrial accident statistics from the Ministry of Employment and Labor of the Republic of Korea revealed that falls, slips, trips, crushing, cutting, and fire accidents occur frequently [45]. Furthermore, the analysis identified the following behavior as the leading direct causes of fatal accidents, in descending order of frequency: leaving unsafe conditions uncorrected > improper use of clothing and protective equipment > insufficient supervision and communication > touching machinery in operation > accessing dangerous areas > improper use of machinery and tools > careless handling of hazardous materials > unsafe postures and movements > disabling safety devices > unsafe speed operation [45]. These types of accidents were taken into consideration during the checklist selection process in this study.

Finally, by integrating the insights from both the experts and the focus group participants, the most appropriate items were selected to finalize the checklist.

FGI are particularly suitable for attitudinal and experiential research and are commonly used to study culture and behavior [46].

The FGI methodology can yield unbiased findings by allowing for the comparison and reflection of multiple perspectives from a group of experts [47].

The questions were divided into two categories on the checklist: risk sensitivity and risk-taking.

The AHP hierarchical analysis method was adopted for this decision [48]. The AHP method is an optimal decision-making methodology that rationally and efficiently makes complex decisions based on the decision-maker’s experience, knowledge, and intuitive judgment [49].

It is a decision-making methodology that determines relative importance in a decision-making situation [50, 51].

In this study, the AHP analysis methodology was employed because it can structure the decision-making in a hierarchical way.

The expert group that applied the AHP method comprised 12 safety experts from the site, academia, and public organizations.

Although this method is similar to the Delphi method, it is characterized by its ability to collect valid opinions without going through the process of repeatedly reviewing expert opinions.

**Fig 1:**
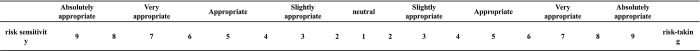
AHP analysis evaluation method.

The AHP method was also employed to quantify the suitability of the questions in accordance with the scale (see Fig 1).

The AHP analysis produced different evaluation results for each evaluator, which were categorized into risk sensitivity and risk-taking.

## 2.1.3 Distinguishing and determining the types of unsafe behavior

The subjects were stratified into five types based on a scale of risk sensitivity and risk-taking.

The types of unsafe behaviors were based on Kazuki’s (1996) classification of behavioral types, and the ’Unacceptable’ zone was added as ’RED ZONE’ according to the ALARP (As Low as Reasonably Practicable) principle of the risk management area.

The ALARP principle is widely applied in safety decision-making, offering a rational approach to risk management [52, 53]. The ALARP principle states that in making decisions that are favorable to health and safety issues, the decision maker, i.e. the worker performing the unsafe act, must implement risk reduction measures [54].

In a study that constructed a behavioral risk model based on the employee behavioral risk of coal mine employees, the ALARP principle was combined to perform an analysis of the risk sensitivity and risk attitude of employees [55].

Based on the ALARP principle, the “unacceptable”, “tolerable”, and “acceptable” types were determined. The minimum number of questions for “unacceptable” was determined by determining the size of the risk according to the question, and the number of questions for “tolerable” and “acceptable” was determined according to the size of the risk.

This was also decided through the opinions of a group of experts. FGI can derive a deeper and broader understanding and meaning of a topic by exploring the experiences and perceptions of participants on the topic selected by researchers [56].

The unsafe behavior checklist is divided into five zones, with risk-taking on the x-axis and risk sensitivity on the y-axis. There are 21 items each for risk sensitivity and risk-taking, with the highest score for each being 21 points. However, scores in the lowest range of 0~5 points for risk sensitivity and the highest range of 16~21 points for risk-taking constitute the dangerous red zone, as the scores are either too low or too high. Scores of 14~21 points for risk sensitivity and 0~8 points for risk-taking make up zone 1, and scores of 6~13 points for risk sensitivity and 0~8 points for risk-taking make up zone 2.

**Fig 2:**
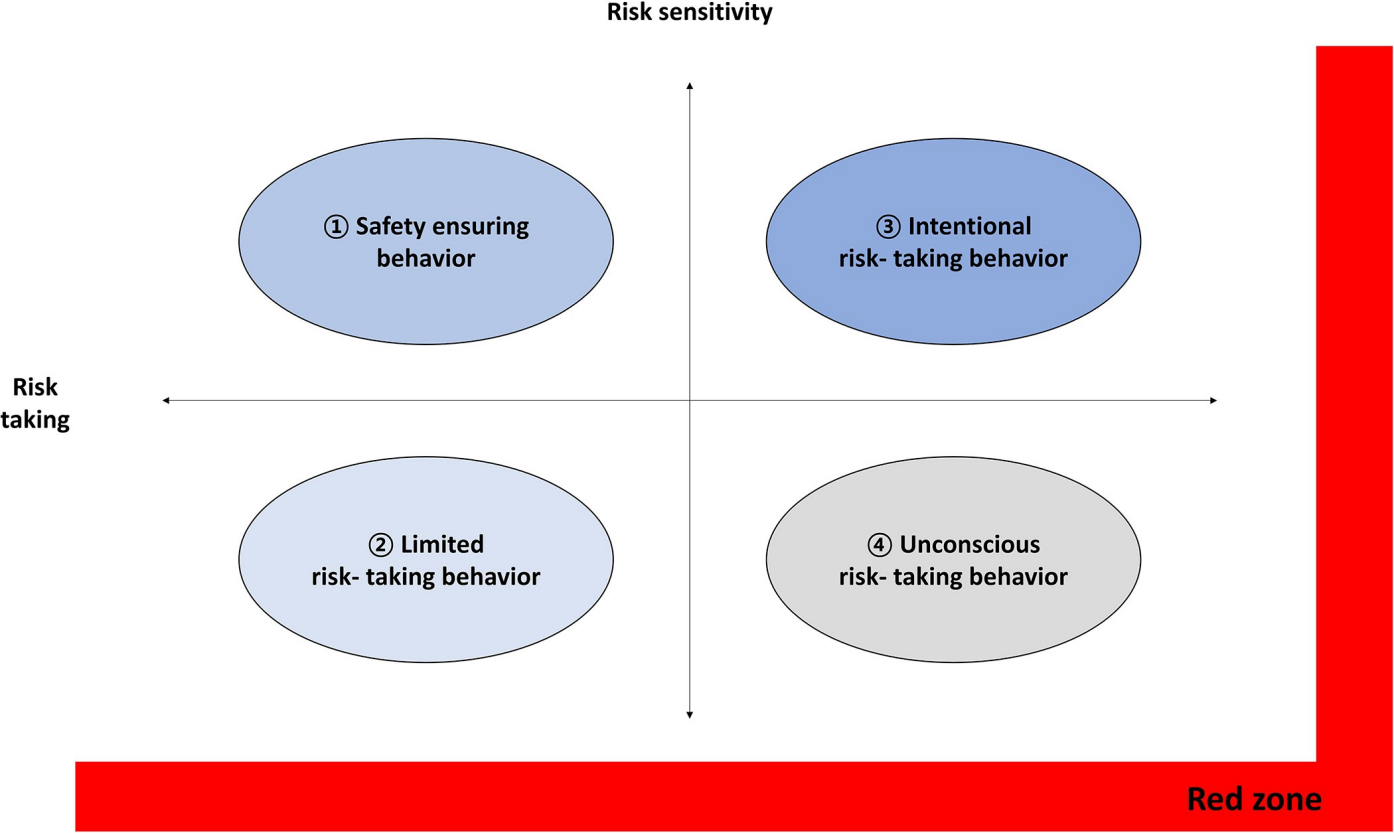
Five type of unsafe behavior [30, 31].

Scores of 14~21 points for risk sensitivity and 9~16 points for risk-taking constitute zone 3, while scores of 14~21 points for risk sensitivity and 6~13 points for risk-taking constitute zone 4(see Fig 2).

Zones 1, 2, 3, and 4 indicate safety ensuring behavior, limited risk-taking behavior, intentional risk-taking behavior, and unconscious risk-taking behavior, respectively, while the red zone indicates unsafe behavior. The safety ensuring behavior indicated in zone 1 is the type of behavior that is characterized by high risk-sensitivity and low risk-taking. In addition, in this behavior, there is a strong tendency to be sensitive to danger and avoid it as much as possible. The limited risk-taking behavior of zone 2 is characterized by both low risk-sensitivity and low risk-taking. Although this type of behavior includes insensitivity to risk, the tendency is to avoid risk, so there is a high probability that safety will be secured as a result. The intentional risk-taking behavior of zone 3 is characterized by both high risk-sensitivity and high risk-taking. Although individuals with this type of behavior have sensitivity to risk, they do not try to avoid risk and instead engage in dangerous situations. The unconscious risk-taking behavior of zone 4 is characterized by low risk-sensitivity and high risk-taking. Individuals with this type of behavior are insensitive to risk and do not try to avoid it.

To work safely, it is very important to increase risk sensitivity and suppress risk-taking. Moreover, despite high sensitivity to risk, high risk-taking can lead to disaster. Therefore, this study aims to identify the types of unsafe behavior by using these risk-sensitivity and risk-taking scores.

**Fig 3:**
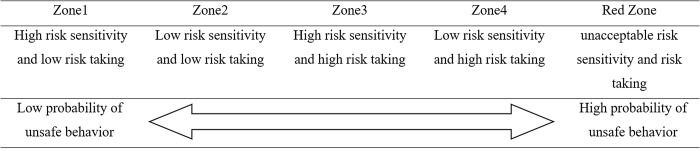
Determining the type of unsafe behavior.

It can be determined that zone 1 has a low type of unsafe behavior, whereas the red zone has a high type of unsafe behavior (See Fig 3).

The risk-sensitivity and risk-taking scores of each individual is assessed using the unsafe behavior checklist.

The scores on the x and y axes is plotted on a graph. The zone where the intersection occurs is determined as the type of unsafe behavior.

### 2.2 Validation of unsafe behavior checklist

#### 2.2.1 Research participants

The survey was conducted targeting general workers and managers working in industrial companies.

The study population for this study comprised randomly selected workers.

Participants were randomly distributed a Google Form with an explanation of how the survey data would be used, and a checklist survey was conducted. The survey was conducted in March–April 2024, and data were collected from 286 participants.

All participants gave consent before taking the survey.

Written consent was obtained from all participants in a Google form survey.

The questionnaire was anonymized.

Participants free to opt out of participation in the study whenever they were uncomfortable.

## 2.2.2 Ethical approvals

The purpose and implications of the study were clearly explained to all participants and were freely explained to those who were willing to participate. Participants who volunteered to participate completed the checklist, which was anonymized.

Review exemption was obtained from the Institutional Review Board of Seoul National University of Science and Technology (No. 2024–0002).

According to the laws of the country where the study was conducted, ethical approval is not needed at the beginning of the work process.

## 2.2.3 Correlation verification

Preliminary testing was conducted with an unspecified sample and the correlation between unsafe behavior and accident experience was validated.

A questionnaire was used to assess risk sensitivity, risk-taking, and accident experience.

Each item was rated with a “Yes” or “No” response. The risk sensitivity and risk-taking aspects of the unsafe behavior checklist are scored as 1 point per correct response to each question.

Accident experience was defined as less than 6 months, 6 months to 1 year, and accident-free, based on the most recent year.

Data were analyzed using SPSS ver. 26.0 (IBM, USA). Phi correlation coefficient and Cramer’s V were used for correlation analysis, and the Kruskal–Wallis test was performed.

The results of the unsafe behavior checklist were classified into unsafe behavior types according to the PSF LICENSE AGREEMENT FOR PYTHON 3.11.2.

The distribution of unsafe behavior types according to the accident experience of the participants was analyzed.

## 3. Results

### 3.1 Determination of unsafe behavior checklist scales using AHP

The AHP analysis produced different evaluation results for each evaluator, which were categorized into risk sensitivity and risk-taking. The mean of the 12 raters’ AHP results was based on a scale of 0.5, where a score below 0.5 was considered risk-taking and a score above 0.5 was considered risk sensitivity. A score of 0.5 was considered “not applicable,” and the question was excluded. (See Table 1).

**Table 1:** Average score and scale classification per question.

Questions	Average	Standard deviation	Division	Questions	Average	Standard deviation	Division
1	0.86	0.02	risk-sensitivity	25	0.29	0.26	risk-taking
2	0.11	0.02	risk-taking	26	0.50	0.24	Not applicable
3	0.85	0.04	risk-sensitivity	27	0.78	0.21	risk-sensitivity
4	0.78	0.19	risk-sensitivity	28	0.13	0.02	risk-taking
5	0.30	0.24	risk-taking	29	0.53	0.17	risk-sensitivity
6	0.88	0.02	risk-sensitivity	30	0.15	0.03	risk-taking
7	0.20	0.20	risk-taking	31	0.62	0.33	risk-sensitivity
8	0.19	0.10	risk-taking	32	0.12	0.01	risk-taking
9	0.50	0.23	Not applicable	33	0.81	0.05	risk-sensitivity
10	0.78	0.21	risk-sensitivity	34	0.17	0.21	risk-taking
11	0.20	0.19	risk-taking	35	0.63	0.24	risk-sensitivity
12	0.70	0.20	risk-sensitivity	36	0.12	0.02	risk-taking
13	0.66	0.22	risk-sensitivity	37	0.24	0.21	risk-taking
14	0.50	0.28	Not applicable	38	0.75	0.20	risk-sensitivity
15	0.22	0.21	risk-taking	39	0.14	0.10	risk-taking
16	0.84	0.10	risk-sensitivity	40	0.17	0.22	risk-taking
17	0.20	0.09	risk-taking	41	0.83	0.20	risk-sensitivity
18	0.76	0.13	risk-sensitivity	42	0.13	0.04	risk-taking
19	0.26	0.22	risk-taking	43	0.80	0.19	risk-sensitivity
20	0.50	0.24	Not applicable	44	0.76	0.16	risk-sensitivity
21	0.87	0.01	risk-sensitivity	45	0.84	0.03	risk-sensitivity
22	0.50	0.23	Not applicable	46	0.85	0.04	risk-sensitivity
23	0.13	0.03	risk-taking	47	0.29	0.24	risk-taking
24	0.80	0.21	risk-sensitivity	48	0.16	0.10	risk-taking

Out of the 48 questions, 6 were excluded as “not applicable,” 21 were selected as “risk sensitivity,” and 21 were selected as “risk-taking” checklist questions.

This is a checklist of determined risk-sensitivities (See Table 2).

**Table 2:** Checklist of risk sensitivity.

No	Question	Check	
1	My safety and health are important to me and my family.	Y	N
2	I think my workplace is less dangerous, and I think that accidents are less likely to occur here than in other workplaces.	Y	N
3	I think it is intimidating to work overtime, work in isolation, or work alone in a place where communication is limited.	Y	N
4	I think safety rules are in place for those with limited experience.	Y	N
5	There are dangerous tasks that used to intimidate me, but not anymore since I have become used to them.	Y	N
6	When something dangerous happens at work, I don’t think I should ask a colleague to handle the situation.	Y	N
7	Although my company and boss emphasize safety excessively, I feel like they consider productivity and quality the top priorities.	Y	N
8	I feel that I want to conduct myself in a safe and exemplary manner at work.	Y	N
9	I think the issue of safety is a personal matter.	Y	N
10	I don’t think I make mistakes.	Y	N
11	If I think there is a risk of me or my colleagues being involved in an accident, I would keep looking into it until it’s all clear.	Y	N
12	If the work content or situation changes during non-routine work, I consider it mandatory. to check the hazards and risks again.	Y	N
13	When a coworker is in an unsafe situation, I will let them know so that they are made aware of the situation immediately.	Y	N
14	My workplace has a strict atmosphere regarding safety, which ensures that no one can commit unsafe acts.	Y	N
15	If I think something is amiss with the safety rules, I communicate about it to my supervisor.	Y	N
16	I know what causes accidents.	Y	N
17	I think facility inspections are sufficient to keep things safe.	Y	N
18	I think it is cool to do the work safely, even if it takes time.	Y	N
19	I do not report near misses sometimes, even when they happen.	Y	N
20	I think the best way to avoid making mistakes due to forgetfulness or distraction is to make sure that one is paying attention.	Y	N
21	I often check incident reports from other workplaces and reflect on my own behavior and work practices.	Y	N

This is a checklist of determined risk-taking (See Table 3).

**Table 3:** Checklist of risk-taking.

No.	Question	Check	
1	I break a rule sometimes when nobody’s watching, such as by overworking or working alone.	Y	N
2	I always make sure my workplace is organized, which means I pick up any rubbish as soon as I see it.	Y	N
3	When something is a hassle, for example, when the right tool is far or the safety switch isn’t nearby, I thoughtlessly take shortcuts.	Y	N
4	If I have a simple task that can be done in a short amount of time, I sometimes proceed without properly shutting down the machine.	Y	N
5	I sometimes run down the stairs or take two or three steps at a time.	Y	N
6	I can work on spur-of-the-moment tasks while remaining calm and aware of the safety of my surroundings and coworkers.	Y	N
7	If my boss or a senior colleague tells me to do something dangerous that breaks the rules, I feel that I have no choice but to do so.	Y	N
8	When I use mechanical equipment, I read the manual before I use it.	Y	N
9	I find myself reaching out without realizing it when things are about to fall to the ground or when I’m about to fall.	Y	N
10	I would follow the rules regarding cell phone use and prohibited conversations (such as while driving a car, in a hospital, train or at a concert).	Y	N
11	I would make sure that any (equipment) inspection is carried out by checking the current condition. (Do not do things like checking a box on a checklist without checking the current status.)	Y	N
12	When I see a coworker breaking the rules, I pretend I didn’t see it because I know I would do the same. I sometimes ignore my coworkers’ rule violations.	Y	N
13	I dispose of my everyday hand tools, supplies, etc., if they are even slightly damaged.	Y	N
14	When I am unsure about something, I can easily ask my boss or coworkers for confirmation.	Y	N
15	If I need to do a task that I think is dangerous, I would contact the authorities, be extra careful, and take safety measures.	Y	N
16	I can honestly report and apologize even if I make a big mistake.	Y	N
17	I often repeat the same mistakes or overlook even minor ones in my daily life and work.	Y	N
18	I don’t use multiple plugs on an electrical outlet.	Y	N
19	I wear protective equipment appropriate for the work environment.	Y	N
20	I sometimes do dangerous things even if I am aware of the danger.	Y	N
21	I have nearly run into people at street corners and so on while walking (running).	Y	N

### 3.2 Statistical analysis

Reliability analysis was conducted using SPSS version 26.0. As the checklist items were dichotomous (yes/no), Kuder-Richardson Formula 20 (KR-20) was employed. The Cronbach’s alpha for the entire set of items was 0.917, indicating high reliability. Furthermore, separate reliability analyses were performed for the 21 items measuring risk-taking propensity and the 21 items measuring risk sensitivity, yielding Cronbach’s alphas of 0.862 and 0.866, respectively. These results suggest that the survey data demonstrate high internal consistency.

Since zone and accident experience were nominal scales, we used the phi correlation coefficient and Cramer’s V for correlation analysis. In the correlation analysis, the phi correlation coefficient was 0.775 (p = 0.000) and Cramer’s V was 0.548 (p = 0.000), indicating a statistically significant correlation between zone classification and accident experience at the p = 0.001 type (See Table 4).

**Table 4:** Correlation analysis result between the zone and accident experience.

		Value	Asymptotic Sig.
Between two nominal scales	Phi	0.775	0.000
	Cramer’s V	0.548	0.000
Number of valid cases		286	

The mean comparison of X-scores for each zone response was performed. Since the groups did not meet normality, a Kruskal–Wallis test was performed, which showed a statistically significant difference between the groups at p = 0.000. Post-hoc analysis revealed no statistically significant difference (p>0.05) for zone 1–zone 2 and zone 3–zone 4. The difference was statistically significant at the p = 0.1 type for zone 4–red zone and at the p = 0.01 type for the remaining responses. (See Table 5).

**Table 5:** Zone comparison by zone correspondence (X-score).

Sample 1-Sample 2	Test Statistics	Standard Error	Standardized test Statistic	Significance Probability	Adjusted Significance Probability^a^
zone1- zone 2	-38.880	20.162	-1.928	0.054	0.538
zone 1- zone 3	-127.787	13.031	-9.806	0.000	0.000
zone 1- zone 4	-141.736	14.503	-9.773	0.000	0.000
zone 1-red zone	-196.099	17.069	-11.489	0.000	0.000
zone 2- zone 3	-88.906	21.904	-4.059	0.000	0.000
zone 2- zone 4	-102.856	22.810	-4.509	0.000	0.000
zone 2-red zone	-157.219	24.522	-6.411	0.000	0.000
zone 3- zone 4	-13.949	16.840	-.828	0.407	1.000
zone 3-red zone	-68.312	19.095	-3.577	0.000	0.003
zone 4-red zone	-54.363	20.128	-2.701	0.007	0.069

a. Significance values were corrected using Bonferroni correction for multiple tests.

The mean comparison of X-scores for each zone response was performed. Since the groups did not meet normality, a Kruskal–Wallis test was performed, which showed a statistically significant difference between the groups at p = 0.000. Post-hoc analysis of the results showed no statistically significant differences (p>0.05) for red zone–zone 4, red zone–zone 2, and zone 2–zone 4. For the other responses, the differences were statistically significant at the p = 0.01 type. (See Table 6).

**Table 6:** Comparison by zone correspondence (Y-score).

Sample 1-Sample 2	Test Statistics	Standard Error	Standardized test Statistic	Significance Probability	Adjusted Significance Probability^a^
red zone—zone 4	10.565	20.112	0.525	0.599	1.000
red zone—zone 2	20.815	24.503	0.849	0.396	1.000
red zone—zone 3	109.554	19.080	5.742	0.000	0.000
red zone—zone 1	161.379	17.056	9.462	0.000	0.000
zone 4- zone 2	10.249	22.792	0.450	0.653	1.000
zone 4- zone 3	98.988	16.827	5.883	0.000	0.000
zone 4- zone 1	150.814	14.491	10.407	0.000	0.000
zone 2- zone 3	-88.739	21.887	-4.054	0.000	0.001
zone 2- zone 1	140.564	20.146	6.977	0.000	0.000
zone 3- zone 1	51.825	13.021	3.980	0.000	0.001

a. Significance values were corrected using Bonferroni correction for multiple tests.

This suggests that the correlation between accident experience and zone classification is reasonable and significant.

### 3.3 Analysis of unsafe behavior type and accident experiences

The data from the questionnaire were compared and analyzed to the type of unsafe behavior according to the accident experience group.

**Fig 4:**
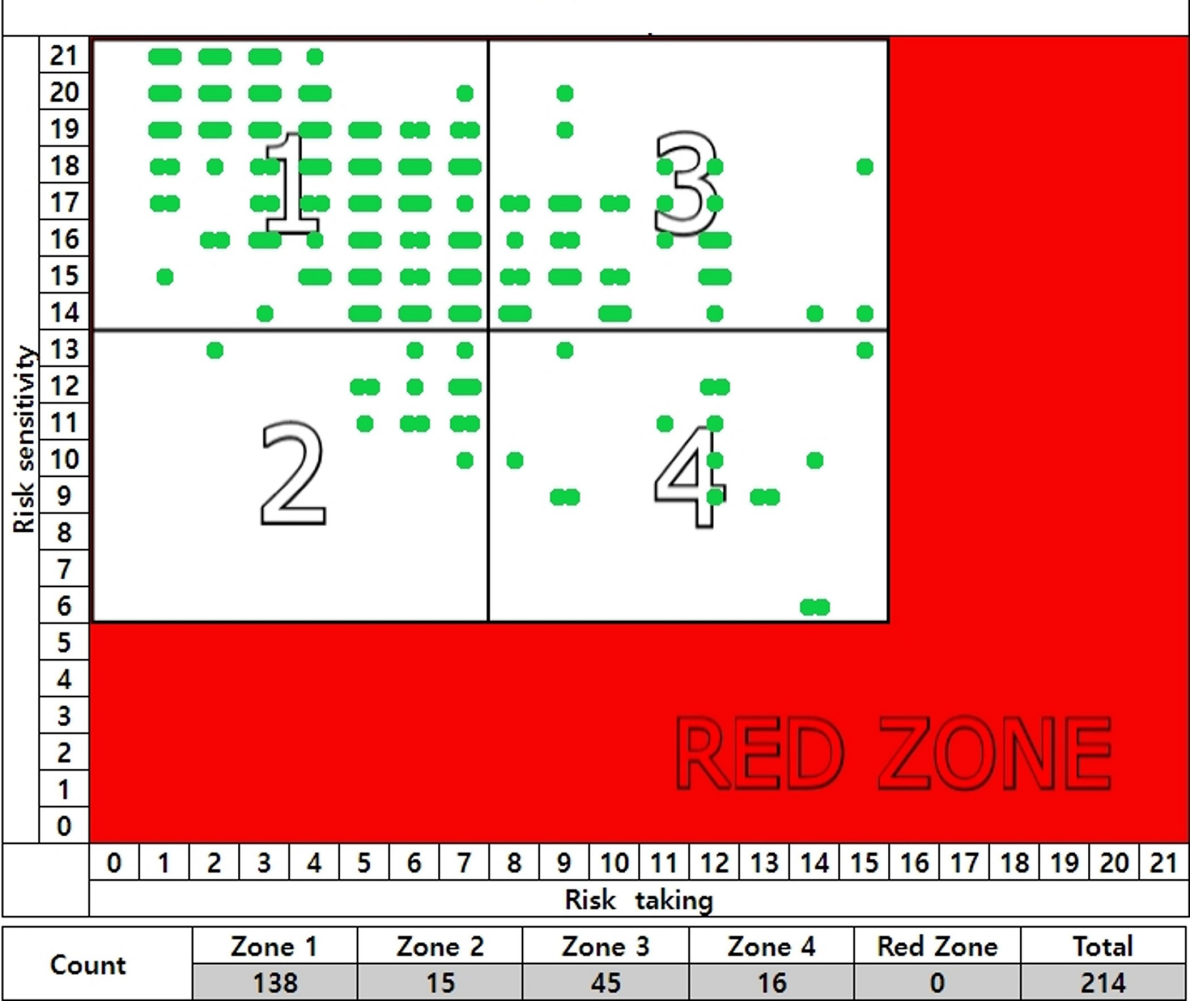
Accident-free group and unsafe behavior.

The 214 accident-free participants (with no accidents during the year) were distributed as follows: 138 in zone 1, 15 in zone 2, 45 in zone 3, 16 in zone 4, and 0 in the red zone. (See Fig 4).

**Fig 5:**
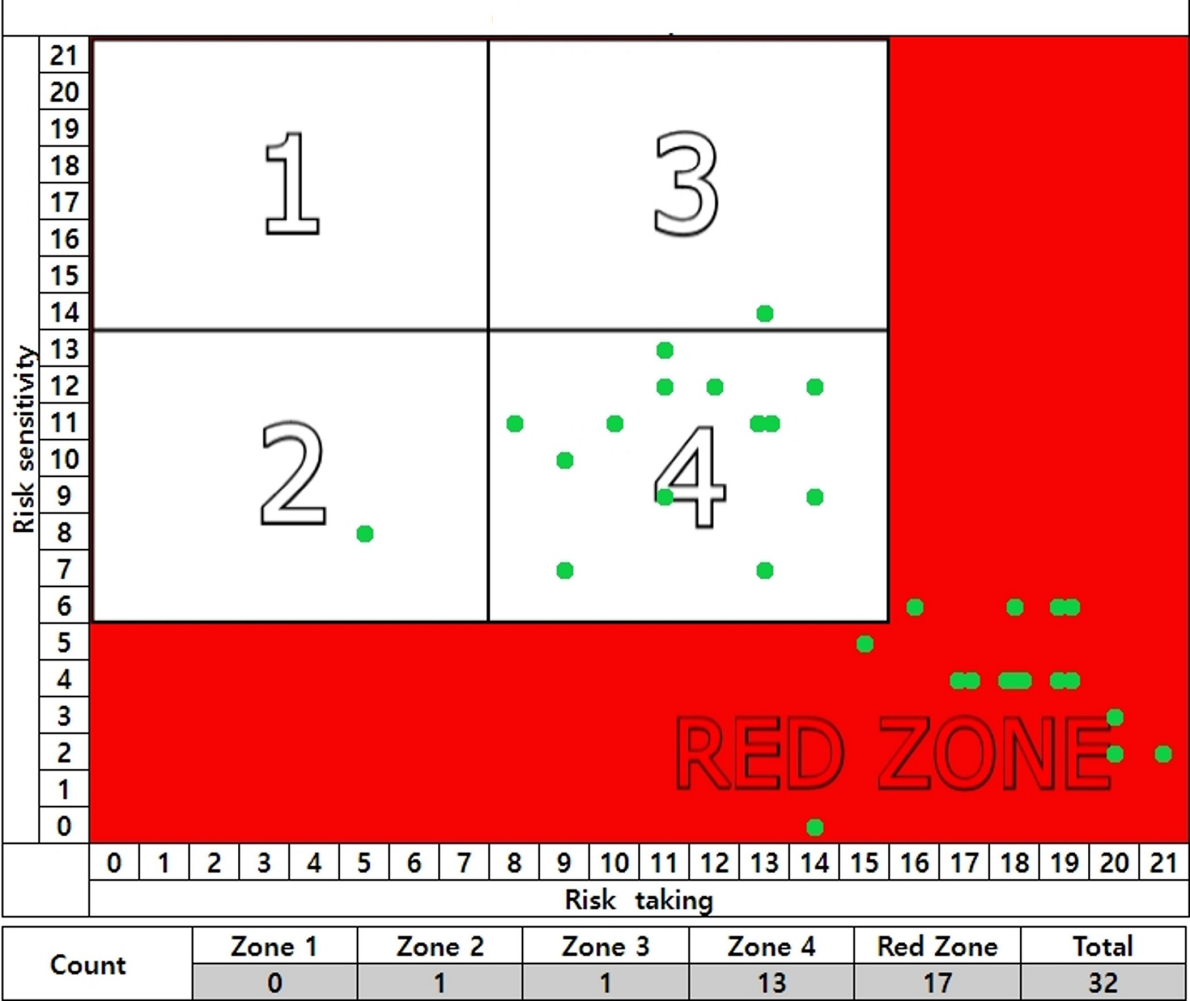
Accident experience group of less than 6 months and unsafe behavior.

Of the 32 participants in the accident experience group of less than 6 months, 0 were in zone 1, 1 was in zone 2, 1 was in zone 3, 13 were in zone 4, and 17 were in the red zone. (See Fig 5).

**Fig 6:**
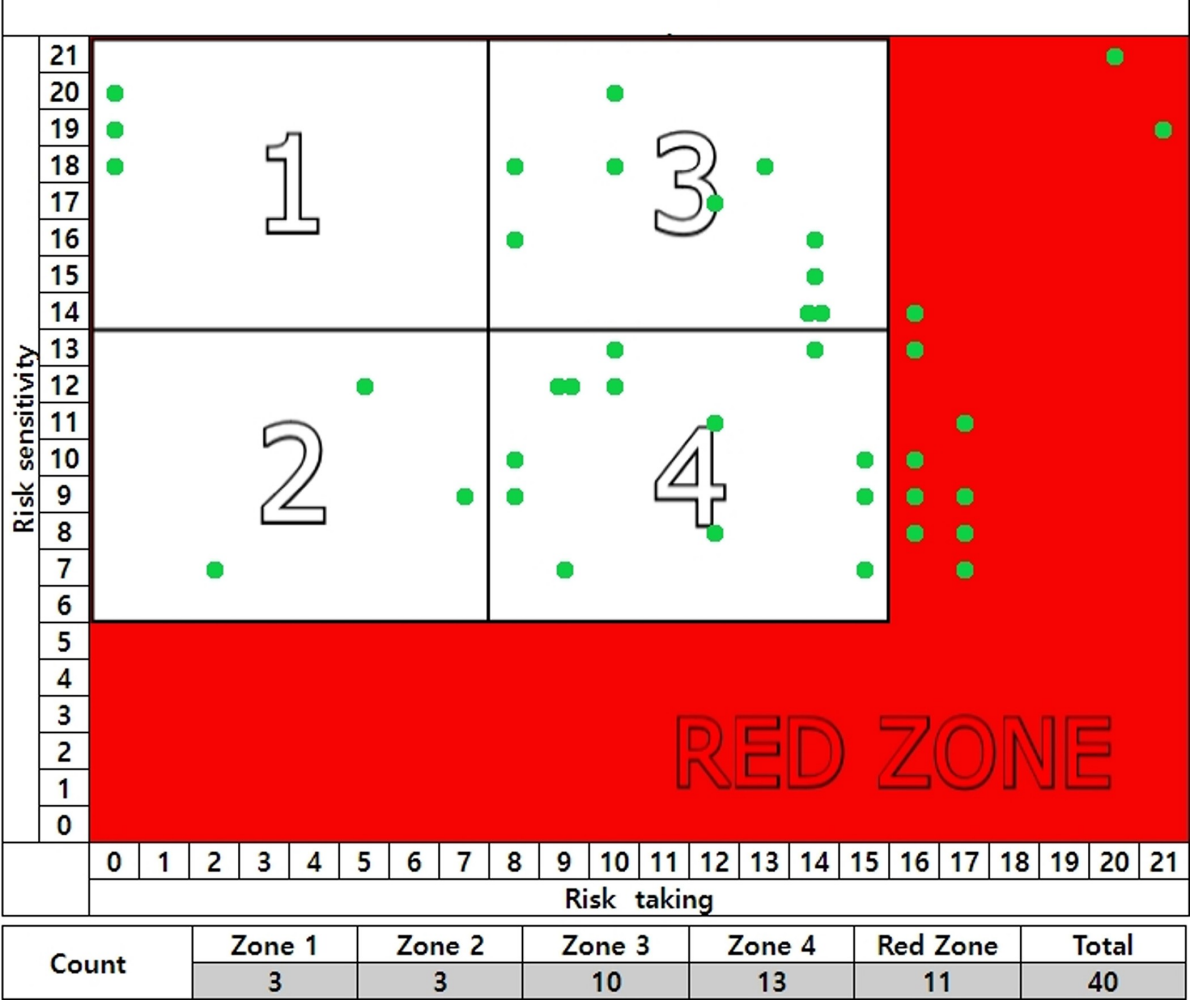
Accident experience group of 6–12 months and unsafe behavior.

Of the 40 participants in the accident experience group of 6–12 months, 3 were in zone 1, 3 were in zone 2, 10 were in zone 3, 13 were in zone 4, and 11 were in the red zone. (See Fig 6).

The accident-free group is 65% likely to engage in safe behavior with low types of unsafe behavior.

In the accident experience group of less than 6 months, more than half (53%) are in the red zone, and 94% of participants have a high percentage of unsafe behavior when zone 4 is included.

Participants experiencing an accident within the last 6–12 months also had high rates of unsafe behavior, as more than 60% were in the red zone and zone 4.

Participants with recent accident experience were more likely to be in the red zone (See Table 7).

**Table 7:** Percentage of zone distribution by accident experience group.

	Accident-free Group	%	Accident Experience Group of Less than 6 Months	%	Accident Experience Group of 6–12 Months	%	Sum
Red zone	0	0	17	53	11	27.5	28
Zone 1	138	65	0	0	3	7.5	141
Zone 2	15	7	1	3	3	7.5	19
Zone 3	45	21	1	3	10	25	56
Zone 4	16	7	13	41	10	32.5	42
Sum	214	100	32	100	40	100	286

## 4. Discussion

Previous studies have shown that most accidents and injuries in the workplace are caused by unsafe behavior of workers, and these studies have examined the factors of unsafe behavior, violation of safety rules, lack of knowledge, and non-use of protective equipment [1]. However, this study developed a checklist to identify the risk behavior type of individual workers based on factors of unsafe behavior from previous studies.

The checklist developed in this study incorporated major accident types and unsafe behavior patterns identified through the analysis of industrial accident statistics in Korea during the question selection process. Furthermore, it covers a wide range of risk factors, making it applicable to industries with similar major risk factors.

The validation results of the unsafe behavior checklist developed in this study revealed that a higher percentage of accident-free workers were distributed in zone 1, “safety ensuring behavior.” In addition, workers who had experienced an accident in the past six months were more likely to be in zone 4, “unconscious risk-taking behavior,” and in the red zone, “unsafe behavior.”

This suggests that the unsafe behavior checklist can be used to predict the type of unsafe behavior of an individual worker.

Similar to how the evaluation of safe driving behaviors in elderly drivers can inform the development of driving competency assessments tailored to specific populations, this study provides foundational data for evaluating unsafe behaviors among industrial workers [24].

The proposed unsafe behavior checklist, akin to tools used by nurses to assess risky behaviors like drug abuse [22], can aid managers in industrial settings in identifying potential unsafe actions among workers. Moreover, it can empower workers to minimize unsafe behaviors and actively enhance their safety practices [57].

Previous research has demonstrated the effectiveness of checklists in improving safety outcomes. For instance, the implementation of surgical safety checklists has been associated with reduced mortality and morbidity rates related to safety-related behaviors in operating rooms [58, 59]. Similarly, a tailored safety behavior checklist for construction sites positively impacted overall safety [59], and behavior-centered feedback derived from such tools led to increased safe work behaviors and decreased injuries [60].

Furthermore, self-assessment strategies, where individuals monitor and evaluate their own actions, have been shown to enhance performance in various contexts, such as improving student instructors’ lesson plan writing skills [61]. It is anticipated that the checklist developed in this study will enable workers to observe and reflect on their own unsafe behaviors, thereby contributing to a reduction in accident risk.

It should be noted, however, that concerns about unsafe behavior focus on the present, and it is difficult to predict future unsafe behavior. This is because it can be misleading to classify workers as high or low type in terms of unsafe behavior based on a single result.

We believe that the unsafe behavior checklist can be a means of predicting the type of unsafe behavior of workers and controlling the unsafe behavior of workers that changes over time.

This study can help predict which workers are likely to engage in safe behaviors and which are likely to engage in unsafe behavior. Workers who are likely to engage in unsafe behavior can be targeted and trained at different types to improve their unsafe behavior. This can help improve unsafe behavior and reduce the frequency of accidents [62].

Accident prevention in the industrial workplace should aim for the early recognition and elimination of all dangers to workers’ lives and health [63].

Therefore, the unsafe behavior checklist developed in this study can be used as an early indicator of the extent to which workers are aware of the danger of occupational accidents at industrial sites, and it can thus be used to prevent accidents.

### 4.1 Strengths and limitations

This study is the first to approach the development of a unsafe behavior checklist through risk-sensitivity and risk-taking of individual workers in an industrial environment.

However, the unsafe behavior checklist study has not yet been fully validated. It is important to acknowledge that the sample used in the study may be unrepresentative, which limits the inferences drawn from the data. Therefore, repeated studies in different sectors are necessary.

## 5. Conclusion

This study’s checklist is expected to facilitate self-observation of unsafe behaviors, thereby contributing to a reduction in accident risk. The checklist’s potential as a tool for evaluating unsafe behaviors has been verified, highlighting its importance in proactively preventing worker accidents.

A risk sensitivity and risk-taking checklist was employed to identify five type of unsafe behavior.

This study concludes that the unsafe behavior checklist can be used to predict accident concerns among workers with high types of dangerous behavior. Therefore, a dangerous behavior checklist is necessary to prevent workers from engaging in unsafe behaviors.

It is expected that the unsafe behavior checklist presented in this study will aid in preventing danger, either by avoiding it or by accepting and controlling it.

In this research, zones were delineated using methods such as FGI, guided by the ALARP principle. Future research should explore the use of more quantitative indicators, such as accident probability, for zone classification.

A single checklist may not sufficiently reflect the unique risk factors of a specific industry. Furthermore, checklists that do not consider industry-specific characteristics can lead to the error of uniformization of unsafe behaviors. Considering these differences, it is necessary to develop detailed items that reflect the characteristics of each industry in the future.

## Supporting information

S1 Checklist (DOCX)

S1 Data (ZIP)
