# Supplementary material for: Development and validation of an unsafe behavior checklist for workers
Source: PLoS One. 2024 Nov 27;19(11):e0314571. doi: 10.1371/journal.pone.0314571 (PMC11602037; doi:10.1371/journal.pone.0314571)
Supplement: S1 Checklist — (DOCX) [file pone.0314571.s001.docx]

Accident Experience

| No. | Question | Check | |
| --- | --- | --- | --- |
| 1 | I have not been involved in an accident of any kind over the past year. | Y | N |
| 2 | I have experienced an accident in the past six months. | Y | N |
| 3 | I have experienced an accident between the last 6 and 12 months. | Y | N |

Risk-sensitivity.

| No | Question | Check | |
| --- | --- | --- | --- |
| 1 | My safety and health are important to me and my family. | Y | N |
| 2 | I think my workplace is less dangerous, and I think that accidents are less likely to occur here than in other workplaces. | Y | N |
| 3 | I think it is intimidating to work overtime, work in isolation, or work alone in a place where communication is limited. | Y | N |
| 4 | I think safety rules are in place for those with limited experience. | Y | N |
| 5 | There are dangerous tasks that used to intimidate me, but not anymore since I have become used to them. | Y | N |
| 6 | When something dangerous happens at work, I don’t think I should ask a colleague to handle the situation. | Y | N |
| 7 | Although my company and boss emphasize safety excessively, I feel like they consider productivity and quality the top priorities. | Y | N |
| 8 | I feel that I want to conduct myself in a safe and exemplary manner at work. | Y | N |
| 9 | I think the issue of safety is a personal matter. | Y | N |
| 10 | I don’t think I make mistakes. | Y | N |
| 11 | If I think there is a risk of me or my colleagues being involved in an accident, I would keep looking into it until it’s all clear. | Y | N |
| 12 | If the work content or situation changes during non-routine work, I consider it mandatory. to check the hazards and risks again. | Y | N |
| 13 | When a coworker is in an unsafe situation, I will let them know so that they are made aware of the situation immediately. | Y | N |
| 14 | My workplace has a strict atmosphere regarding safety, which ensures that no one can commit unsafe acts. | Y | N |
| 15 | If I think something is amiss with the safety rules, I communicate about it to my supervisor. | Y | N |
| 16 | I know what causes accidents. | Y | N |
| 17 | I think facility inspections are sufficient to keep things safe. | Y | N |
| 18 | I think it is cool to do the work safely, even if it takes time. | Y | N |
| 19 | I do not report near misses sometimes, even when they happen. | Y | N |
| 20 | I think the best way to avoid making mistakes due to forgetfulness or distraction is to make sure that one is paying attention. | Y | N |
| 21 | I often check incident reports from other workplaces and reflect on my own behavior and work practices. | Y | N |

Risk-taking

| No. | Question | Check | |
| --- | --- | --- | --- |
| 1 | I break a rule sometimes when nobody’s watching, such as by overworking or working alone. | Y | N |
| 2 | I always make sure my workplace is organized, which means I pick up any rubbish as soon as I see it. | Y | N |
| 3 | When something is a hassle, for example, when the right tool is far or the safety switch isn’t nearby, I thoughtlessly take shortcuts. | Y | N |
| 4 | If I have a simple task that can be done in a short amount of time, I sometimes proceed without properly shutting down the machine. | Y | N |
| 5 | I sometimes run down the stairs or take two or three steps at a time. | Y | N |
| 6 | I can work on spur-of-the-moment tasks while remaining calm and aware of the safety of my surroundings and coworkers. | Y | N |
| 7 | If my boss or a senior colleague tells me to do something dangerous that breaks the rules, I feel that I have no choice but to do so. | Y | N |
| 8 | When I use mechanical equipment, I read the manual before I use it. | Y | N |
| 9 | I find myself reaching out without realizing it when things are about to fall to the ground or when I’m about to fall. | Y | N |
| 10 | I would follow the rules regarding cell phone use and prohibited conversations (such as while driving a car, in a hospital, train or at a concert). | Y | N |
| 11 | I would make sure that any (equipment) inspection is carried out by checking the current condition. (Do not do things like checking a box on a checklist without checking the current status.) | Y | N |
| 12 | When I see a coworker breaking the rules, I pretend I didn’t see it because I know I would do the same. I sometimes ignore my coworkers’ rule violations. | Y | N |
| 13 | I dispose of my everyday hand tools, supplies, etc., if they are even slightly damaged. | Y | N |
| 14 | When I am unsure about something, I can easily ask my boss or coworkers for confirmation. | Y | N |
| 15 | If I need to do a task that I think is dangerous, I would contact the authorities, be extra careful, and take safety measures. | Y | N |
| 16 | I can honestly report and apologize even if I make a big mistake. | Y | N |
| 17 | I often repeat the same mistakes or overlook even minor ones in my daily life and work. | Y | N |
| 18 | I don’t use multiple plugs on an electrical outlet. | Y | N |
| 19 | I wear protective equipment appropriate for the work environment. | Y | N |
| 20 | I sometimes do dangerous things even if I am aware of the danger. | Y | N |
| 21 | I have nearly run into people at street corners and so on while walking (running). | Y | N |

Risk-sensitivity Risk-taking

| NO | Check | |  | NO | Check | |  |
| --- | --- | --- | --- | --- | --- | --- | --- |
| 1 | Y | N |  | 1 | Y | N |  |
| 2 | Y | N |  | 2 | Y | N |  |
| 3 | Y | N |  | 3 | Y | N |  |
| 4 | Y | N |  | 4 | Y | N |  |
| 5 | Y | N |  | 5 | Y | N |  |
| 6 | Y | N |  | 6 | Y | N |  |
| 7 | Y | N |  | 7 | Y | N |  |
| 8 | Y | N |  | 8 | Y | N |  |
| 9 | Y | N |  | 9 | Y | N |  |
| 10 | Y | N |  | 10 | Y | N |  |
| 11 | Y | N |  | 11 | Y | N |  |
| 12 | Y | N |  | 12 | Y | N |  |
| 13 | Y | N |  | 13 | Y | N |  |
| 14 | Y | N |  | 14 | Y | N |  |
| 15 | Y | N |  | 15 | Y | N |  |
| 16 | Y | N |  | 16 | Y | N |  |
| 17 | Y | N |  | 17 | Y | N |  |
| 18 | Y | N |  | 18 | Y | N |  |
| 19 | Y | N |  | 19 | Y | N |  |
| 20 | Y | N |  | 20 | Y | N |  |
| 21 | Y | N |  | 21 | Y | N |  |
| Total |  | |  | Total |  | |  |
|  |  | |  |  |  |  |  |

| R  I  S  K  -S  E  N  S  I  T  I  V  I  T  y | 21 |  |  |  |  |  |  |  |  |  |  |  |  |  |  |  |  |  |  |  |  |  |  |
| --- | --- | --- | --- | --- | --- | --- | --- | --- | --- | --- | --- | --- | --- | --- | --- | --- | --- | --- | --- | --- | --- | --- | --- |
|  | 20 |  |  |  |  |  |  |  |  |  |  |  |  |  |  |  |  |  |  |  |  |  |  |
|  | 19 |  |  |  |  |  |  |  |  |  |  |  |  |  |  |  |  |  |  |  |  |  |  |
|  | 18 |  |  |  |  |  |  |  |  |  |  |  |  |  |  |  |  |  |  |  |  |  |  |
|  | 17 |  |  |  |  |  |  |  |  |  |  |  |  |  |  |  |  |  |  |  |  |  |  |
|  | 16 |  |  |  |  |  |  |  |  |  |  |  |  |  |  |  |  |  |  |  |  |  |  |
|  | 15 |  |  |  |  |  |  |  |  |  |  |  |  |  |  |  |  |  |  |  |  |  |  |
|  | 14 |  |  |  |  |  |  |  |  |  |  |  |  |  |  |  |  |  |  |  |  |  |  |
|  | 13 |  |  |  |  |  |  |  |  |  |  |  |  |  |  |  |  |  |  |  |  |  |  |
|  | 12 |  |  |  |  |  |  |  |  |  |  |  |  |  |  |  |  |  |  |  |  |  |  |
|  | 11 |  |  |  |  |  |  |  |  |  |  |  |  |  |  |  |  |  |  |  |  |  |  |
|  | 10 |  |  |  |  |  |  |  |  |  |  |  |  |  |  |  |  |  |  |  |  |  |  |
|  | 9 |  |  |  |  |  |  |  |  |  |  |  |  |  |  |  |  |  |  |  |  |  |  |
|  | 8 |  |  |  |  |  |  |  |  |  |  |  |  |  |  |  |  |  |  |  |  |  |  |
|  | 7 |  |  |  |  |  |  |  |  |  |  |  |  |  |  |  |  |  |  |  |  |  |  |
|  | 6 |  |  |  |  |  |  |  |  |  |  |  |  |  |  |  |  |  |  |  |  |  |  |
|  | 5 |  |  |  |  |  |  |  |  |  |  |  |  |  |  |  |  |  |  |  |  |  |  |
|  | 4 |  |  |  |  |  |  |  |  |  |  |  |  |  |  |  |  |  |  |  |  |  |  |
|  | 3 |  |  |  |  |  |  |  |  |  |  |  |  |  |  |  |  |  |  |  |  |  |  |
|  | 2 |  |  |  |  |  |  |  |  |  |  |  |  |  |  |  |  |  |  |  |  |  |  |
|  | 1 |  |  |  |  |  |  |  |  |  |  |  |  |  |  |  |  |  |  |  |  |  |  |
|  | 0 |  |  |  |  |  |  |  |  |  |  |  |  |  |  |  |  |  |  |  |  |  |  |
|  | | 0 | 1 | 2 | 3 | 4 | 5 | 6 | 7 | 8 | 9 | 10 | 11 | 12 | 13 | 14 | 15 | 16 | 17 | 18 | 19 | 20 | 21 |
|  |  | Risk-taking | | | | | | | | | | | | | | | | | | | | | |
